# Supplementary material for: Towards unified AI-driven fracture mechanics: the extended deep energy method (XDEM)
Source: Nat Commun. 2026 Aug 15;17:8492. doi: 10.1038/s41467-026-76748-1 (PMC13484471; doi:10.1038/s41467-026-76748-1)
Supplement: Supplementary file 3 [file 41467_2026_76748_MOESM3_ESM.zip › pic/Results/Stress_intensity_factor/Cross/XDEM/angle_45/crack_error_sy_pred.pdf]

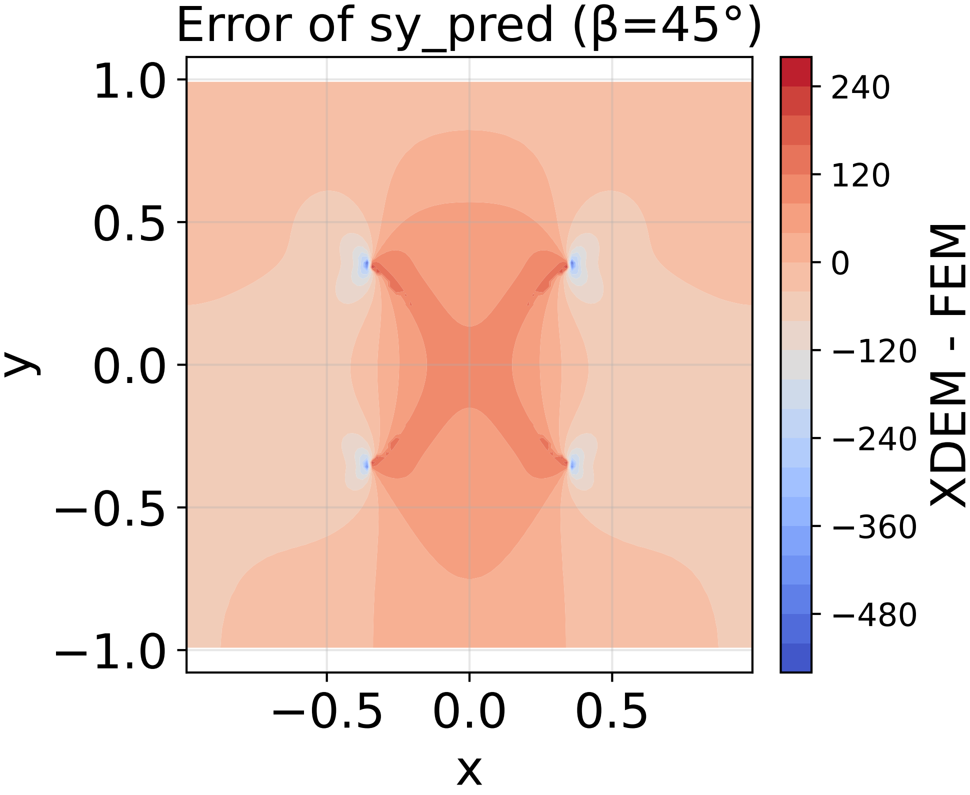

Error of sy_pred (β=45°)
1.0
0.5
0.0
−0.5
−1.0
y
−0.5
0.0
0.5
x
240
120
0
−120
−240
−360
−480
XDEM - FEM
